# Nonlinear optical characterization of copper oxide nanoellipsoids

**DOI:** 10.1038/s41598-019-47941-8

**Published:** 2019-08-06

**Authors:** Ganjaboy S. Boltaev, Rashid A. Ganeev, P. S. Krishnendu, Ke Zhang, Chunlei Guo

**Affiliations:** 1 The Guo China-US Photonics Laboratory, State Key Laboratory of Applied Optics, Changchun Institute of Optics, Fine Mechanics and Physics, Chinese Academy of Sciences, Changchun, 130033 China; 2 The Institute of Optics, University of Rochester, Rochester, NY 14627 USA

**Keywords:** Structural properties, Nanoparticles

## Abstract

Recently, nonspherical nanoparticles took attention due to advanced properties of these structures. We report the study of the nonlinear optical properties of copper oxide nanoellipsoids using 800 nm and 400 nm, 60 fs pulses. The optical limiting effect of copper oxide nanoellipsoids is analyzed. The influence of band gap of copper nanoparticles and copper oxide nanoellipsoids on their nonlinear optical response was studied. For the first time, the low- and high-order nonlinear optical responses of copper nanoellipsoids were studied. The magnitudes of nonlinear optical parameters of the suspension of copper oxide nanoellipsoids were measured to be *γ* = 1.23 × 10^−15^ cm^2^ W^−1^, and *β* = 1.0 × 10^−11^ cm W^−1^ respectively. We observed the four-fold enhancement of the nonlinear optical refraction of copper oxide nanoellipsoids at the wavelength of 400 nm, 60 fs probe pulses compared to 800 nm radiation. We also analyzed the high-order nonlinear response of CuO nanoellipsoids through generation of high-order harmonics of 800 nm, 60 fs pulses in the plasmas produced during laser ablation of the nanoellipsoid-contained targets. We demonstrated the harmonics up to the 35th order (E = 50 eV) in case of single-color pump and 24th (30 eV) in case of two-color pump.

## Introduction

The nanostructures of transition metal oxides have attracted significant attention in recent years due to their mechanical, electrical, optical and magnetic properties^1^. Analysis of electronic structures in transition metals has shown that appearance of defect states affects the band gap of the nanoparticles^2^. In most cases the chemical methods were used for synthesis of the uniform nanostructures of transition metal oxides^3,4^. Meanwhile, laser ablation is also useful for preparation of nanoparticles from bulk metal or semiconductor targets^5^. Particularly, the influence of surrounding liquid media and the ablating conditions resulted in fabrication of Cu nanoparticles (NPs) in acetone and CuO NPs in chloroform was reported by Gondal *et al*.^6^. The influence of virgin coconut oil to the sizes of Cu NPs was demonstrated by Sadrolhosseini, *et al*.^7^. It was shown that the increase of ablation time of copper in suspension of virgin coconut oil led to the decrease of the sizes of NPs.

The investigation of the nonlinear optical properties of metal oxides, such as Co_3_O_4_, V_2_O_5_, CuO, Fe_2_O_3_, Mn_3_O_4_, Cr_2_O_3_ have shown larger nonlinear responses compared to non-oxidized nanostructures^8,9^. Among the advantages of the metal oxides are the large optical nonlinearities (10^−8^–10^−7^ esu), good thermal and chemical stability as well as mechanical strength. In particular, the copper oxide nanostructures have the largest figure of merit based on the ratio of the magnitude of the optical nonlinearity to the linear optical absorption. The nonlinear optical properties of spherical copper nanoparticles doped in indium tin oxide matrix by ion implantation were studied by Ryasnyansky *et al*.^10^. Third-order susceptibility of copper nanoparticles was estimated by Huang *et al*.^11^. The *χ*^(3)^/*α*_0_ values for copper colloids were obtained to be of the magnitude of 10^−12^ to 10^−11^ esu cm. Meanwhile, no comparative analysis of the influence of band gaps on the nonlinear optical properties of CuO nanostructures was reported.

The studies of laser-produced plasmas (LPP) created on the surfaces of solid copper target using femtosecond and picosecond laser pulses for generation of low- and high-order harmonics was reported in refs^12–15^. Those studies revealed that the harmonic yields from the plasmas produced by picosecond and femtosecond pulses of the same fluence were comparable, despite the significant difference in the intensities of the heating pulses. The component of the plasma can cause constructive or destructive action on the overall nonlinear optical response of a whole ensemble of plasma particles. The decrease of conversion efficiency of high harmonic generation (HHG) generally was caused by some components (e.g. free electrons, quantum dots or aggregated nanoparticles and microparticles) of plasma plumes evaporated during laser ablation using ultrashort laser pulses. This behavior was caused due to the nature of these particles, which reached the interaction area with probe femtosecond pulses. In our studies we took the attention for analysis of the high-order nonlinear response of CuO nanoellipsoids (NEs) via generation of high-order harmonics.

In this paper, we study the synthesis of Cu NPs and self-aggregated CuO NEs produced during laser ablation. We analyze the change of the band gap of oxidized Cu NPs in water. This process leads to formation of CuO NEs with 2.4 eV band gap due to “bottom-up” aggregation of Cu NPs in deionized water. We analyze an influence of the band gaps of Cu NPs and CuO NEs on their nonlinear optical properties. The enhancement of the nonlinear refraction index of suspension and the red-shifted band gap of CuO NEs is observed. We also analyze the high-order nonlinear response of CuO nanostructures via HHG in laser-produced plasmas containing these species.

## Results and Discussion

### The Morphology and Linear Optical Responses of the Samples

**Figure 1:**
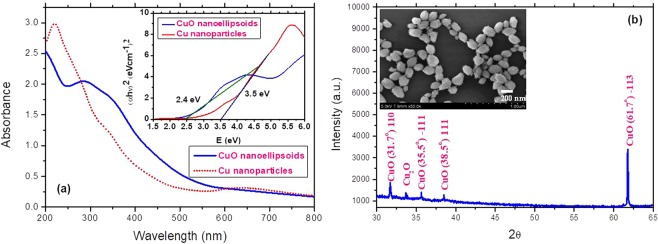
(**a**) Absorption spectra of Cu nanoparticles during laser ablation using 200 ps, 800 nm pulses and self-aggregated CuO nanoellipsoids from Cu nanoparticles in the deionized water. (**b)** The XRD of CuO nanoellipsoids. Inset: SEM of CuO nanoellipsoids.

Prior to carrying the study of third order optical nonlinearities of CuO NEs we provided analysis the morphological and linear absorptive characteristics of Cu NPs and CuO NEs. In the case of Cu NPs the broad surface plasmon resonance (SPR) peaks was observed at 620 nm that was attributed to the presence of spherical nanoparticles (Fig. 1(a)), similarity to the results of ref.^16^. The green color of Cu NPs suspension was attributed to the strong absorption near the infrared range of spectrum. In the case of CuO NEs, the broad surface plasmon resonance (SPR) peak was observed at 300 nm, which is a characteristic of these nanoellipsoids in deionized water. The increase in the band gap of the CuO NEs points out the quantum confinement effect arising due to presence of the small nanoparticles and nanoellipsoids.

The volume ratio of small-sized nanostructures is the most important parameter in the quantum confinement effect. The red-shift of plasmonic peaks shows the growth of the sizes of nanostructures. In the meantime, the ratio of surface area to volume for a materials or substances containing nanoparticles has a significant effect on the optical properties of the material. It means that this ratio increases with a decrease of the radius of the spheres. The number of carriers increases with the blue-shift of SPR. The enhancement of third-order nonlinearity can be explained by a high concentration of the carriers on the surfaces of CuO nanoellipsoids.

We calculated the band gaps based on the Tauc’s model. The Tauc’s model allows determining the band gap energies of Cu nanoparticles and CuO nanoellipsoids by extrapolation of the absorption spectra measured by spectrophotometer. Though the Tauc’s plot extrapolation has been widely adopted for extracting bandgap energies of semiconductors, there is a lack of theoretical consideration for applying it for the nanocrystals. The optical transitions in CuO NEs have been formulated based on a purely theoretical approach. For α-Fe_2_O_3_, α-Cu_2_V_2_O_7_, and monoclinic-BiVO_4_ phases, the Automated Tauc Analysis methodology has been developed in ref.^17^. We used this methodology for extrapolation of the absorption spectra of CuO NEs to estimate the band gaps of these materials.

The optical band gaps of samples were estimated by fitting the equation

1 $${\alpha }_{0}h\nu =C{(h\nu -{E}_{g})}^{1/2}$$

with the linear absorption spectra (see inset in Fig. 1(a)). Here *h*, *v* and C are the Planck’s constant, frequency and constant, respectively. The band gaps of Cu NPs and CuO NEs were defined to be 3.5 and 2.4 eV.

The presence of CuO NEs was confirmed using the X-ray diffraction spectroscopy (Fig. 1(b)). The atomic ratio of Cu to O was 1:1. The XRD of CuO nanoellipsoids has earlier been analyzed in ref.^18^, and showed the crystalline structure of these species. In our studies, the diffraction patterns were observed at 2θ = 31.7 (110), 35.49 (−111), 38.68 (111), and 61.45° (−123), which were assigned to the reflection lines from monoclinic CuO NEs. Our results were found to be in agreement with the reported diffraction patterns of CuO NEs from above refereed paper, thus confirming the crystalline structure of our NEs.

The inset in Fig. 1(b) shows the SEM of CuO NEs. The sizes of the orthogonal axes of these NEs were 150 and 200 nm. We analyzed the statistics of the sizes of CuO NEs using SEM and TEM of these nanostructures. The majority of NEs had the sizes, which were equal to *a* = 200 nm *b* = 150 nm. The ellipticity of CuO NEs was equal to 0.75. The dispersion of sizes was 40 nm by *a* axis and 30 nm by *b* axis. The ellipticity of NEs did not change during the change of the sizes of NEs. The ellipticity of these particles was in the range of 0.7–0.75, which is relatively similar to the ellipsoidal particles.

The morphology of CuO nanoellipsoids was studied during three days after laser ablation of Cu target in water. For the first day we observed the small-sized Cu nanoparticles, which caused the variation of the absorption spectra of fresh ablated samples. After three days the morphology of Cu NPs was changed. The synthesized CuO NEs were attached to each other to form the small CuO nanocrystals, which were identified by XRD peaks (Fig. 1b). The most important parameters of nanostructures are their dimensions. In the case of self-aggregated metal oxide nanoparticles their sizes are distributed in a broad range. For example, the dimensional parameters of such self-aggregated CuO nanostructures were analyzed in ref.^19^.

**Figure 2:**
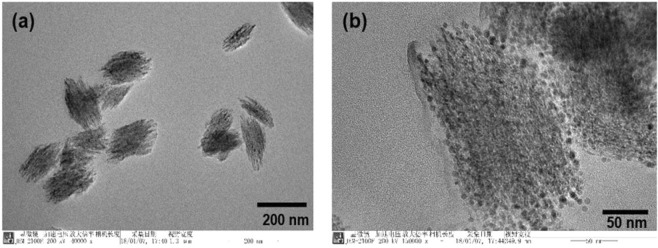
TEM images of self-aggregated CuO NEs in water after laser ablation of bulk copper target using 800 nm, 200 ps heating pulses: **(a)** 200 nm scale, **(b)** 50 nm scale.

We also analyzed the TEM of CuO NEs (Fig. 2(a)). The formation of copper nanoellipsoids can be explained by the “bottom-up” approach^20^. The aggregation based on this mechanism results in formation of the single crystalline assemblies of CuO NEs containing a few hundred quantum dots. We observed the small monocrystalline structures, which aggregated during oxidation of Cu NPs in deionized water (Fig. 2(b)). The formation of ellipsoids-like particles was observed (with the width of these particles in the range of 120–180 nm and the length 200–250 nm). The TEM images of these particles revealed the simultaneous presence of some grain-like particles (Fig. 2b), which were undetected in the SEM micrographs. The TEM image of single NE clearly showed the roughness of the surface around the edges thus suggesting that these seeds were formed by the accumulation of other smaller sized particles.

### The Third-Order Nonlinear Optical Responses of the Samples

**Figure 3:**
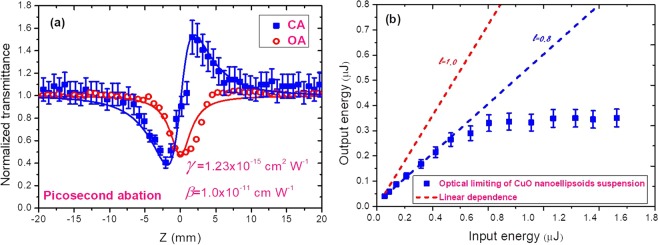
(**a**) Z-scans using open-aperture (OA) and closed-aperture (CA) schemes. The Cu NEs were synthesized using ablation by the 800 nm, 200 ps pulses. **(b)** Optical limiting curve of self-aggregated CuO NEs suspension with the same linear transmittance of 80% at 800 nm.

The Z-scan method was used for the determination of the magnitude of the nonlinear optical parameters of Cu NPs and CuO NEs using the 60 fs fundamental (λ = 800 nm) pulses, and its second harmonic generation (λ = 400 nm) from Ti:S laser. Figure 3 shows the Z-scans of CuO NEs suspension and the optical limiting (OL) in this suspension using the 800 nm, 60 fs probe pulses. In case of CuO NEs suspension the positive nonlinear refractive properties were observed in the field of femtosecond pulses with 1 kHz repetition rate. (Fig. 3(a), filled squares).

**Figure 4:**
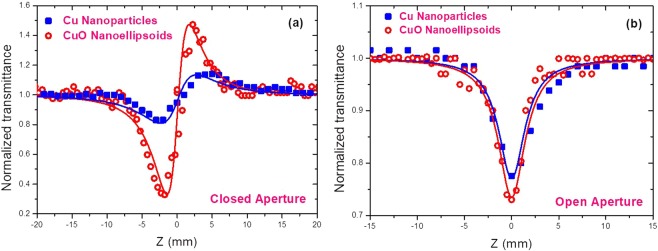
Z-scans of copper NPs and copper oxide NEs suspensions (wavelength 400 nm, pulse duration 60 fs): (**a**) CA, and (**b**) OA Z-scans.

The magnitudes of the nonlinear optical parameters of the CuO NEs at λ = 800 nm were determined to be *γ* = 1.2 × 10^−15^ cm^2^ W^−1^, and *β* = 1.0 × 10^−10^ cm W^−1^ (Fig. 3(a)). Open aperture (OA) Z-scan allowed analyzing the optical limiting properties of CuO NEs suspension at the wavelength of 800 nm, 60 fs pulses. The suspension was contained in 1-mm thick quartz cuvette. A linear transmittance of CuO NEs suspension was equal to 80%. Figure 4(b) shows OL in CuO NEs suspension that was attributed to the two-photon absorption (2PA) measured by using the Z-scans (Fig. 3(a), empty circles). The linear part of this dependence between output energy and input energy of 800 nm, 60 fs pulses in the OL curve was observed up to the energies of ~ 0.6 *μ*J (Fig. 3(b)). The deviation from linearity of this curve occurs with further growth of the input pulse energy from 0.6 to 1.6 μJ. The pure water was also studied in this energy range of propagated laser pulses and did not show the OL properties. The slope of linear fitting for pure deionized water at different input pulse energies was equal to 1.0, while the slope of linear fitting of CuO NEs at small energies was equal to 0.8. Further decrease of output energy was attributed to OL due to 2PA. We did not observe the OL in the Cu NPs using 800 nm, 60 fs probe pulses at the same range of pulse energies as in case of CuO NEs.

The comparative analysis of the nonlinear optical response of Cu NPs and CuO NEs suspensions was performed using the 400 nm radiation. Figure 4 shows the CA and OA Z-scan dependences of these suspensions. The nonlinear optical parameters of samples, measured using 400 nm, 60 fs probe pulses, are presented in Table 1. The four-fold increase of nonlinear refractive index in these two suspensions can be associated with the decrease of the band gap of CuO NEs. Detailed explanation of this behavior was given by Sheik-Bahae *et al*.^21^. The band gap and refractive index are related with each other as follows^22^:

2 $${n}^{4}{E}_{g}=95\,eV$$

**Table 1:** Nonlinear optical characteristics of the Cu nanoparticles and CuO nanoellipsoids.

Sample	Band gap (eV)	800 nm, 60 fs			400 nm, 60 fs		
		*γ* (cm^2^ W^−1^)	*β*_2PA_ (cm W^−1^)	*χ*^(3)^ (esu units)	*γ* (cm^2^ W^−1^)	*β*_2PA_ (cm W^−1^)	*χ*^(3)^ (esu units)
Cu NPs	3.5	—	—	—	1.52 × 10^−15^	3.5 × 10^−11^	6.0 × 10^−10^
CuO NEs	2.4	1.23 × 10^−15^	1.0 × 10^−11^	1.2 × 10^−9^	6.0 × 10^−15^	3.8 × 10^−11^	2.8 × 10^−9^

According to this relation, the refractive index of a semiconductor can be determined by knowledge the energy band gap (*E*_*g*_). This relation is based on the general assumption that all energy levels in a solid are scaled down by a factor of 1/${\varepsilon }_{eff}^{2}$, where *ε*_eff_ is the effective dielectric constant of the samples. The relation between the 2PA coefficient and band gap was analyzed by Van Stryland *et al*.^23^. It was shown that the 2PA varies as ${E}_{g}^{-3}$. This dependence can be represented by the following equation:

3 $${\beta }_{2PA}=K\sqrt{{E}_{p}}F(2h\nu /{E}_{g})/{n}^{3}{E}_{g}^{3}$$

where *K* is a material-independent constant, *n* is the linear refractive index, and ${E}_{p}=8{P}^{2}m/{h}^{2}$ (where *P* is the Kane momentum parameter and *m* is the electron mass) is nearly material independent for a wide variety of semiconductors. This equation permits a prediction of 2PA coefficients of materials at different wavelengths. The function *F*, whose exact form depends on the assumed band structure, is a function only of the ratio of the photon energy *hv* to *E*_*g*_, which determines the states that are optically coupled. In our case we demonstrated the variation of 2PA coefficient due to different band gap energies of Cu NPs and CuO NEs. The values of *β*, *γ*, and *χ*^(3)^ calculated from the experimental data are presented in Table 1.

The large third-order optical nonlinearities of spherical nanoparticles of the Cu_2−x_S for strong localized surface plasmon resonances (LSPR) absorption bands in the near infrared region were investigated by Hamanaka *et al*.^24^. The resonantly enhanced nonlinear susceptibility was observed in the vicinity of the LSPR peak, similar to the case of noble metal nanoparticles. The aim of our work was to shown the variation of the third-order optical nonlinearities depending on the band gaps of Cu NPs and CuO NEs. We compared the third-order nonlinear optical parameters of our samples with Cu_2−x_S nanoparticles and showed the enhancement of the third-order nonlinearity of these nanostructures due to the influence of LSPR. In our case of 400 nm, 60 fs probe pulses the large nonlinear susceptibilities of CuO NEs were obtained (see Table 1). We also compared the nonlinear optical parameters of CuO NEs and Cu NPs at the same conditions. Our aim was to show the advantages of CuO NEs compared with Cu NPs at the same wavelength of exciting laser radiation.

We carried out the pump-probe measurements to study the temporal response of the CuO NEs using 400 nm, 60 fs pulses. During these studies we did not observe the temporal response of the transient absorption profile of CuO NEs. Earlier, the transient absorption spectroscopy using an above-bandgap pump beam and below-bandgap probe beam to analyze the relaxation and recombination dynamics of the CuO nanocrystals at various pump fluencies was reported in ref.^25^. They used the pulses of 1550 nm (1.6 eV) radiation as the probe beam and 780 nm (0.8 eV) radiation as the pump beam. In the meantime, the band gap of 50 nm CuO NPs was 1.5 eV. The three time constants were reported in those studies. The first time constant varied with pump fluence from 330 fs to 630 fs, and was attributed to momentum relaxation via carrier-carrier scattering in the valence band as well as exciton-exciton annihilation. The second time constant was unchanged at 2 ps and was attributed to the energy relaxation via carrier-phonon scattering within the valence band.

In our case, the probe and pump beam were higher than band gap of CuO NEs. We also used as a probe beam the 800 nm, 60 fs pulses and second harmonic generation of Ti:S laser at the 400 nm, 60 fs as a pump beam, however, did not observed the temporal variations of the nonlinear optical response of NEs.

The third-order nonlinear response analyzed in refs^26,27^ is based on the thermal effect. High repetition rate of probing pulses led to the accumulative thermal effects, which resulted in the formation of the thermal lens. In our case, we observed the nonlinear refraction based on the Kerr nonlinearities depending on the band gaps of Cu NPs and CuO NEs. We demonstrated the advantages of CuO NEs compared with Cu NPs.

The main difference between the CuO NEs and CuO NPs is that CuO NEs have a 3D ellipsoidal shape, while CuO NP-contained nanosheets have a 2D sheet shape. 3D nanomaterials have the tunable properties, while 2D nanomaterials possess the exceptional inherent properties. During our study, we observed the variation of the properties of self-aggregated CuO NEs originated from Cu NPs. In the meantime the 3D shape of CuO NEs can play important role during the studies of the high-order nonlinearities of these species. We analyzed HHG in the plasmas containing CuO NEs.

### The High-Order Nonlinear Response of CuO NEs via HHG

**Figure 5:**
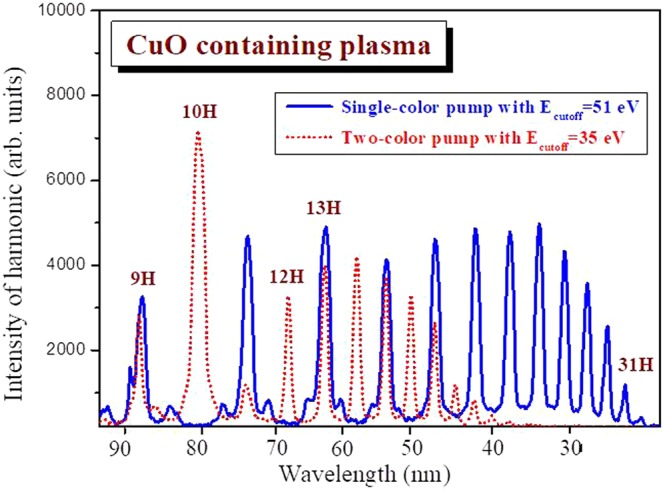
Spectra of harmonics obtained using single-color (solid blue line) and two-color (dashed red line) pumps of CuO NEs containing plasma. Odd and even harmonics were generated in the CuO containing plasma plumes using 800 nm and 400 nm, 60 fs pulses as the driving pulses. Plasma plumes were produced on the surfaces of bulk Cu target using 800 nm, 200 ps heating pulses. Correspondingly, the highest order harmonic with cutoff energy (51 eV) and (35 eV) in the cases of single–color pump and two-color pump schemes were obtained.

The highest order nonlinear optical properties of CuO NEs were analyzed using high-order harmonic generation of 800 nm, 60 fs laser pulses. The plasma plumes containing CuO NEs were created using 800 nm 200 ps heating pulses at vacuum conditions. The same fluence of heating ablation pulses, which was applied during formation of CuO NEs and nanoparticles, was used for creation of laser-produced plasma plumes. In Fig. 5, the spectra of high-order harmonic generation (HHG) are presented.

The analysis of harmonic spectra generated in CuO plasma plumes allows concluding about the attractive properties of CuO NPs as the emitters of coherent extreme ultraviolet radiation. We compared our results with early studies of HHG in copper plasma^15^. The plasma contained singly-charged ions and atoms of Cu during ablation using pulses of different duration. Harmonic generation up to the 17^th^ order was reported in those studies. Meanwhile, in our case, when the plasma contained CuO nanostructures, the harmonics up to the 31^st^ order were observed, which was attributed to the influence of small-sized quantum dots with larger recombination cross-section^28,29^.

## Conclusions

In summary, the Cu NPs and CuO NEs were synthesized during laser ablation process of bulk copper target in deionized water using 800 nm, 200 ps pulses. The self-aggregation of Cu NPs to the CuO NEs led to the change of the band gaps of these nanostructures. Our nonlinear optical studies of these suspensions have shown that the nonlinear refractive properties of CuO NEs depend on the band gap. We observed the increase of the nonlinear refractive index by decreasing the band gap of CuO nanostructures. The nonlinear refractive index and nonlinear absorption coefficient of suspensions at the wavelength of 800 nm, 60 fs probe pulses were 1.23 × 10^−15^ W cm^−1^ and 1.0 × 10^−11^ W cm^−1^. We also analyzed the high-order nonlinear response of CuO NEs through high-order harmonic generation of 800 nm, 60 fs pulses in the plasma containing ablated CuO NEs from thin films. We demonstrated the high harmonic up to 35th order (E = 50 eV) in the case of single-color pump and 24th order (30 eV) in case of two-color pump.

## Methods

### Synthesis of CuO NEs from Cu NPs in the water

**Figure 6:**
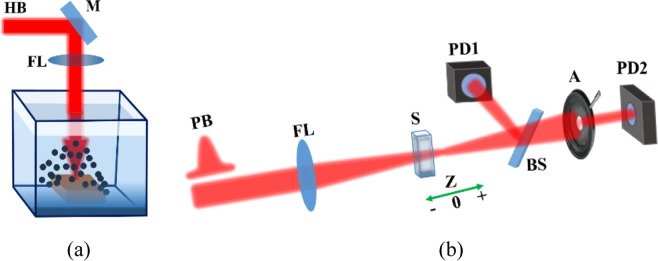
(**a**) Laser ablation of copper target in liquids: HB, heating beam; M, mirror; FL, focusing lens. **(b)** Z-scan scheme: PB, probe beam, FL, focusing lens; S, sample; BS, beam splitter, PD1 and PD2, photodiodes; A, aperture.

The CuO NEs was synthesized from ablated Cu target in water using picosecond pulses (Spitfire Ace, Spectra Physics). The output characteristics of the uncompressed pulses of this laser system were as follows: wavelength λ = 800 nm, pulse duration t = 200 ps, 1 kHz pulse repetition rate. The bulk Cu target with sizes 10 × 10 × 2 mm^2^ was immersed in a 20 mm thick quartz cell filled with deionized water. The focusing lens with 100 mm focal length was used for focusing laser radiation on the surface of bulk copper target [Fig. 6(a)].

The radiation fluence on the surface of ablated target was measured to be 30 J cm^−2^. The Cu was ablated during 30 min. The volume ratio of synthesized Cu NPs in suspension was estimated to be 1 × 10^−4^. The target was moved to maintain the efficiency of laser ablation on the fresh surface of Cu target. The suspension was rinsed during ablation to decrease the absorption of laser radiation in front of the surface of copper target. No surfactants were added in the suspension to prevent the aggregation of nanoparticles. Copper nanoparticles were studied using TEM and SEM. The optical absorption of Cu NPs suspension was analyzed by spectrometer (Agilent Technology). The oxidation of ablated suspension occurred during ablation. The stability of CuO NEs was controlled during the nonlinear optical studies of suspension. We also analyzed the morphology of Cu NPs in water a days after laser ablation.

### Z-scan technique

The investigation of the nonlinear optical characteristics of nanoparticle suspension in water was performed at the wavelengths of 800 nm and 400 nm (t = 60 fs) using the Z-scan technique^30^. The suspensions containing Cu NPs and CuO NEs in a 2 mm thick quartz cells was moved along the Z-axis through the focal plane of 400 mm focal length lens using a translation stage controlled by a computer (Fig. 6(b)).

By defining the relative coordinate *x* = *z/z*_0_, *z*_0_ being the Rayleigh length, the dependence of the normalized transmittance *T(x)* in the case of closed-aperture (CA) Z-scan can be written as^31^

4 $$T(x)=1-\frac{4x}{({x}^{2}+9)({x}^{2}+1)}{\rm{\Delta }}{\Phi }_{0}+\frac{2({x}^{2}+3)}{({x}^{2}+9)({x}^{2}+1)}{\rm{\Delta }}{\Psi }_{0}$$

Here, *z*_0_ = 0.*5kw*_*o*_^2^, *ΔΦ*_0_ = *kγL*_*eff*_*I*_0_, Δ*Ψ*=*βI*_*0*_*L*_*eff*_/2, *k* = *2π/λ* is the wave number, *w*_*o*_ is the beam waist radius of the focused radiation, *I*_0_ is the intensity of probe beam at the focal plane of focusing lens, *γ* is the nonlinear refractive index, *β* is the nonlinear absorption coefficient, *L*_*eff*_ = [*1*−*exp* (−*α*_0_*L*)]/*α*_0_ is the effective length of nonlinear medium, *L* is the sample thickness and *α* is the linear absorption coefficient of suspension. The nonlinear refraction index and nonlinear absorption coefficient were determined by theoretical fitting of experimental data using Eq. (4). The error bars for determination of the absolute values of nonlinear absorption and refraction coefficients were estimated to be ± 25% due to uncertainty in the measurements of the beam waist of focused probe beam.

### High-order harmonic generation in laser-produced plasmas

**Figure 7:**
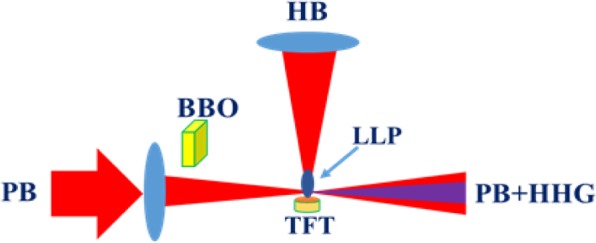
Experimental setup of HHG in LPP: PB; Probe beam, BBO; nonlinear crystal, HB; heating beam, LLP; laser-produced plasmas, TFT; thin film target, PB + HHG; probe beam and high harmonic generation converted from probe beam in laser-produced plasmas.

The high-order harmonic generation was performed at the vacuum conditions (Fig. 7). Here we present our experimental arrangements for HHG in laser-produced plasmas. We used 800 nm, 60 fs laser pulses as the probe pulses, which were converted to the harmonics in the plasmas created on the surface of the thin film containing CuO NEs using 800 nm, 200 ps heating pulses. More details about the experimental setup of HHG in the LPP of thin films were reported in ref.^32^. The thin film target on the surfaces of glass substrate was prepared from CuO suspension using spin-coating machine at the normal conditions. The uncompressed picosecond heating pulses were used for ablation of CuO NEs. The ablated CuO NEs entered the interaction area with probe femtosecond pulses (800 nm, 30 fs, 1 kHz) after 70 ns delay time. The delay time between heating and probe pulses was adjusted using optical delay line. The position of the target was adjusted using the translating stage to achieve the maximal harmonic yield generated in LPP. The extreme ultraviolet spectrometer containing a cylindrical mirror and a 1200 grooves/mm flat field grating with variable line spacing allowed detecting the harmonic up to 71th order. The spectra of harmonics were recorded by a micro-channel plate with phosphor screen, and the harmonics were imaged by a CCD camera.
